# DNA polymerase ζ has robust reverse transcriptase activity relative to other cellular DNA polymerases

**DOI:** 10.1016/j.jbc.2024.107918

**Published:** 2024-10-23

**Authors:** Ryan Mayle, William K. Holloman, Michael E. O’Donnell

**Affiliations:** 1 Howard Hughes Medical Institute and the Department of DNA Replication, The Rockefeller University, New York, New York, USA; 2 Department of Microbiology & Immunology, Weill Cornell Medicine, New York, New York, USA

**Keywords:** DNA polymerase zeta, reverse transcriptase, translesion DNA polymerase, double-strand DNA break repair, DNA synthesis

## Abstract

Cell biology and genetic studies have demonstrated that DNA double-strand break (DSB) repair can be performed using an RNA transcript that spans the site of the DNA break as a template for repair. This type of DSB repair requires a reverse transcriptase to convert an RNA sequence into DNA to facilitate repair of the break, rather than copying from a DNA template as in canonical DSB repair. Translesion synthesis (TLS) DNA polymerases (Pol) are often more promiscuous than DNA Pols, raising the notion that reverse transcription could be performed by a TLS Pol. Indeed, several studies have demonstrated that human Pol η has reverse transcriptase activity, while others have suggested that the yeast TLS Pol ζ is involved. Here, we purify all seven known nuclear DNA Pols of *Saccharomyces cerevisiae* and compare their reverse transcriptase activities. The comparison shows that Pol ζ far surpasses Pol η and all other DNA Pols in reverse transcriptase activity. We find that Pol ζ reverse transcriptase activity is not affected by RPA or RFC/PCNA and acts distributively to make DNA complementary to an RNA template strand. Consistent with prior *S. cerevisiae* studies performed *in vivo*, we propose that Pol ζ is the major DNA Pol that functions in the RNA-templated DSB repair pathway.

DNA damage plagues all cell types. Damage comes in the form of any alteration in DNA structure leading to a change in coding capacity or deterioration of genome integrity. Different types of radiation, genotoxic cancer therapies, environmental toxins, and endogenous oxidation products and metabolites can introduce an enormous range of chemical changes in DNA structure. Self-induced damage can be incurred by collisions and aberrant action of endogenous protein complexes that replicate, transcribe, and alter the topology of DNA. To cope with such a vast array of damage, cells have an arsenal of countermeasures to restore genomic integrity. This includes an assortment of mechanical systems to traverse or remove damaged bases, restore tracts of damaged nucleotides, and rejoin broken DNA molecules. With certain notable exceptions of direct reversal of DNA damage by photoreactivation of ultraviolet light-induced cyclobutane dimers (1) and demethylation of alkylated bases *via* dioxygenases or suicide demethylases (2), a common feature of all DNA repair mechanisms is the requirement for some amount of DNA repair synthesis. This could range from the incorporation of a mere few nucleotides in the case of excision repair of a single modified base (3), to hundreds of nucleotides in the case of DNA double-strand break (DSB) repair (4), or even thousands in the case of break-induced replication (5).

Specialized DNA polymerases exist to repair short gaps created after base excision (3), traverse damaged nucleotides in concert with ongoing DNA replication (6, 7), to blunt or polish broken DNA ends in preparation for rejoining by non-homologous end joining (8) or to extend across a break during repair *via* microhomology-mediated end joining. Repair of DSBs by the homologous recombination (HR) pathway requires longer tracts of synthesis and is thought to utilize the replicative DNA Pol δ in a migrating D-loop reaction that provides part of the means for bridging the broken DNA ends (9). In HR the conventional template used for repair synthesis is an undamaged homologous DNA partner, usually the sister chromatid (10).

Evidence has emerged from recent studies suggesting that RNA transcripts might also be directly utilized as templates in DSB repair (11, 12). In this case, DNA synthesis would arise from a reverse transcriptase (RT) activity. In studies of RNA templated repair performed in *Saccharomyces cerevisiae*, reverse transcriptase activity was in some cases attributed to that encoded by the Ty element retrotransposon (13). However, this is believed to function through a cDNA intermediate, where the RNA is only indirectly used as a repair template (14, 15). An overview of this pathway is diagramed in Fig. S1. Pol θ of mammalian cells has also been shown to harbor a robust reverse transcriptase activity (16) and is required for DSB repair by the microhomology-mediated end-joining pathway (17). Recent biochemical studies have shown that Pol η has some reverse transcriptase capability (18) and can promote RNA-templated repair *in vivo* (19). New genetic investigations in yeast have suggested that Pol ζ performs the RT activity necessary for RNA-templated repair (13). In contrast to the cDNA-mediated Ty RT repair pathway, these newly identified repair pathways are proposed to involve repair synthesis directly templated by RNA. An example of such a repair mechanism is illustrated in Figure 1, where a transcript mRNA is used to bridge a DNA DSB and facilitate repair *via* RT synthesis.

**Figure 1:**
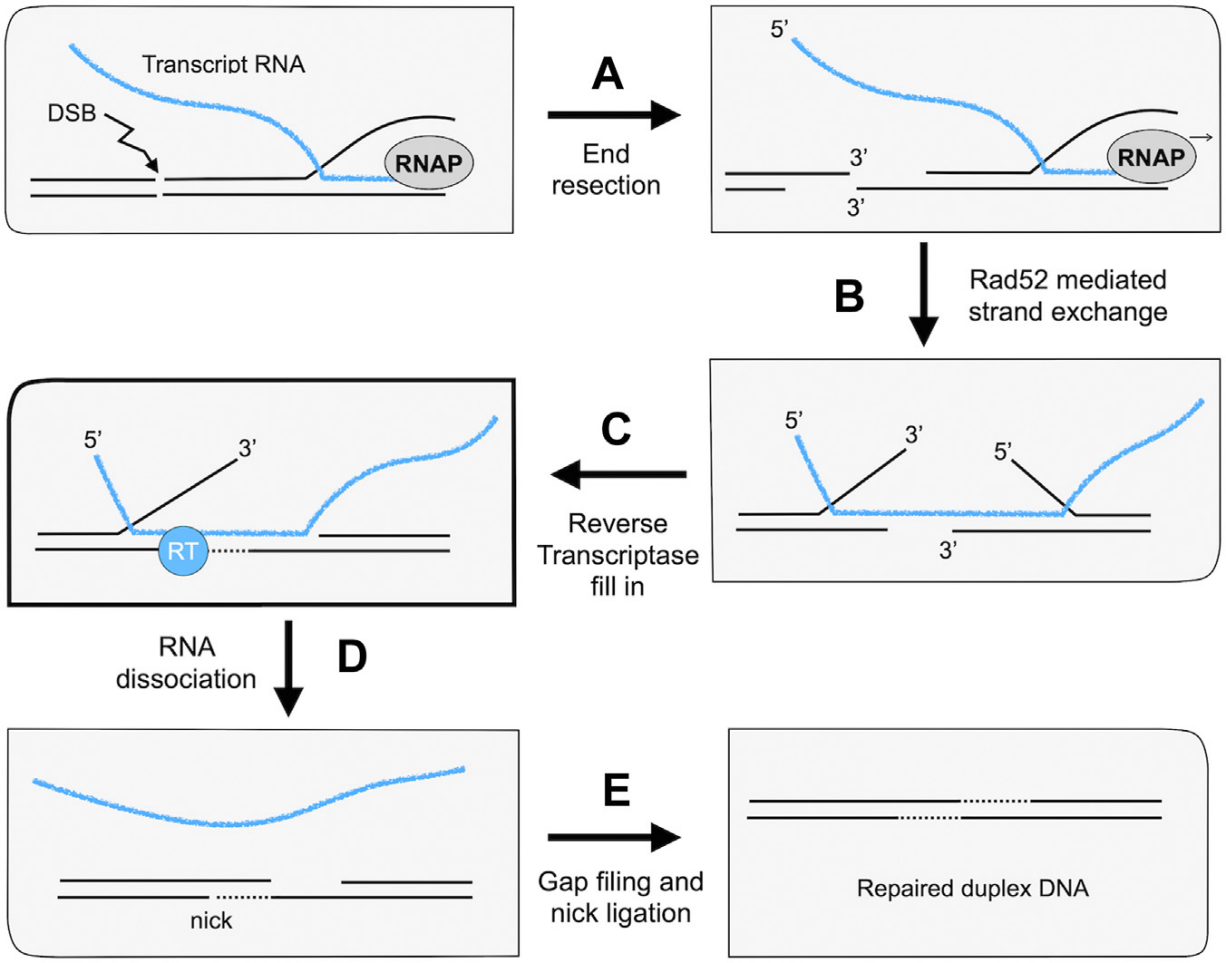
**Model of RT activity for RNA mediated double strand DNA break repair.***A*, dsDNA break may occur after transcription has produced an mRNA transcript. In this event, any resection and consequent loss of DNA information can be “rescued” by the mRNA transcript as follows. Following (*A*) end resection, (*B*) the mRNA can bridge the DSB *via* annealing and Rad52-mediated strand exchange. After this, (*C*) the gap created by resection can be filled in by a reverse transcriptase. After (*D*) the RNA dissociates from the break site, (*E*) normal DNA templated gap filling and nick ligation restore an intact duplex.

To help address the gap in knowledge about DNA Pol RT activity in this important field, we have purified all seven nuclear DNA Pols of budding yeast and compared their ability to perform reverse transcription. We find that Pol ζ clearly contains the most proficient RT activity. Although comparison of extension by Pol ζ on RNA compared to DNA templates shows greater activity on DNA, Pol ζ′s RT activity is more robust than any of the other DNA Pols, and is not hindered by RPA, RFC, or PCNA. Thus, the results of this report suggest that Pol ζ may be the major enzyme involved in the RNA-directed DSB repair pathway, consistent with cellular studies of this pathway in budding yeast (13).

## Results

**Figure 2:**
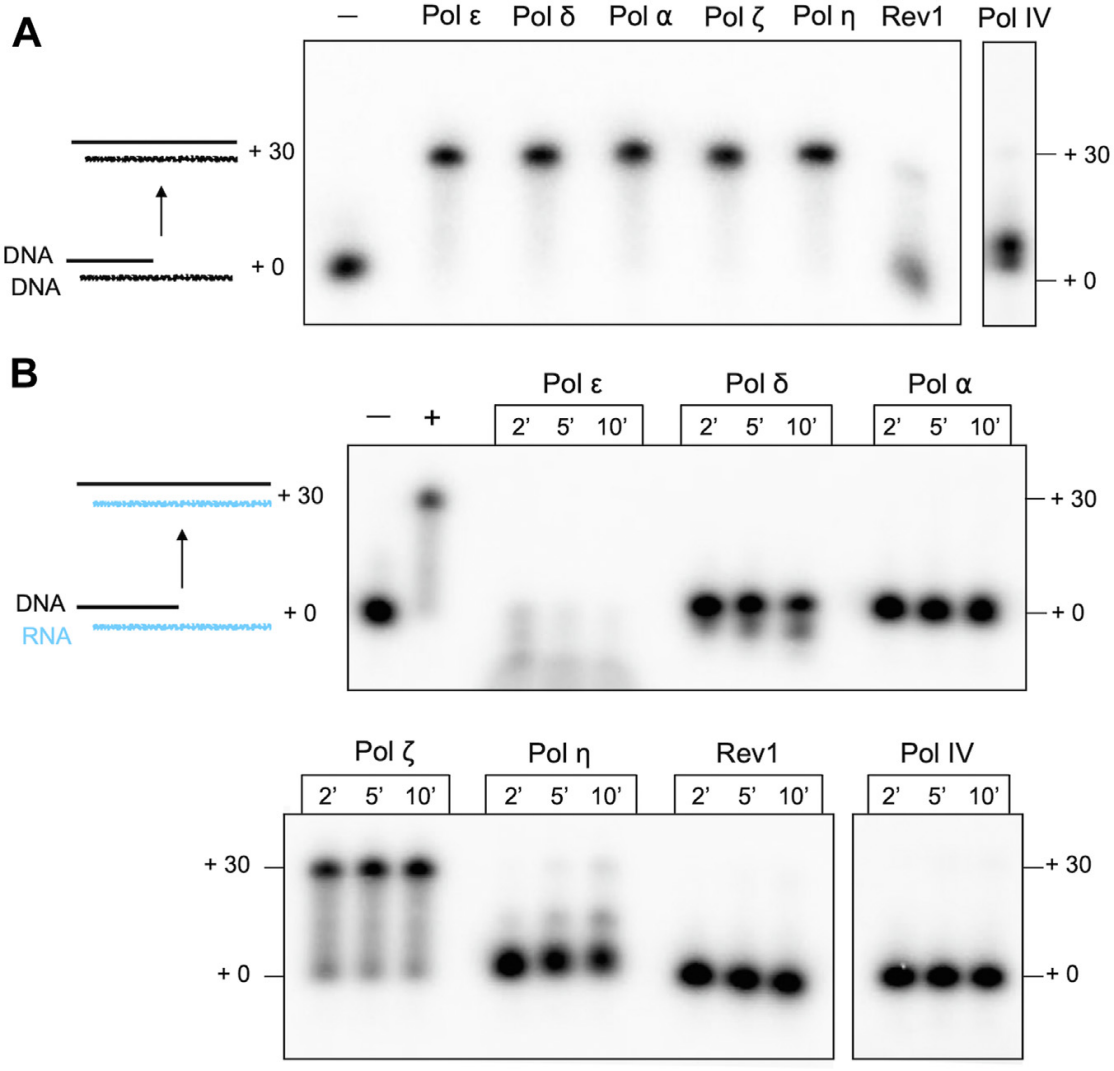
**Pol ζ is the only yeast Pol having significant Reverse Transcriptase activity.***A*, comparative DNA synthesis activity using 20 nM of either Pol ε, δ, ⍺, ζ, or η. Elevated concentrations were used for Rev1 (60 nM) and Pol IV (100 nM) in order to observe any extension activity. *B*, RT activity of each Pol, using the same fmol of Pol as in panel *A*.

We have purified all seven of the nuclear DNA Pols of *S. cerevisiae* (Fig. S2) and have tested them for relative DNA polymerase and reverse transcriptase (RT) activity. These include the replicative polymerases Pol α, δ and ε, the translesion polymerases Pol η, ζ, and Rev1, and the X-family polymerase Pol IV, considered the equivalent of metazoan Pol β, which is dedicated to base excision repair (20). As shown in Figure 2*A*, these DNA Pols can efficiently utilize dNTPs to extend a primer across a 30 nt ssDNA template strand, with the exception of Rev1 and Pol IV. Rev1 was previously characterized in many systems to be a very specialized Pol, and typically only incorporates a single dC nucleotide (21). The activity of Pol IV is less well characterized than other yeast polymerases, but it was shown to fill short gaps and act distributively to extend at longer single strand regions (22, 23, 24).

This report tests these DNA Pols for RT compared to DNA polymerase activity using a primed substrate with comparable RNA or DNA template strands. The results show that all the Pol preparations have DNA Pol activity, although Rev1 shows minimal extension activity as expected (Fig. 2*A*). Upon examination of RT activity using an RNA template strand of the same sequence of the DNA template, we observe that only Pol ζ has significant RT activity (Fig. 2*B*). Note, however, that Pol η does have some RT activity, consistent with previous reports (18, 25). However, at equal molar concentrations Pol η has substantially weaker RT activity than Pol ζ. Pol ε, and to a lesser extent Pol δ, degrades the primer rather than extending it when utilizing an RNA template strand. We do not rule out the possibility that Pol δ and Pol ⍺ may extend a nucleotide or two on RNA, as was previously reported (12).

**Figure 3:**
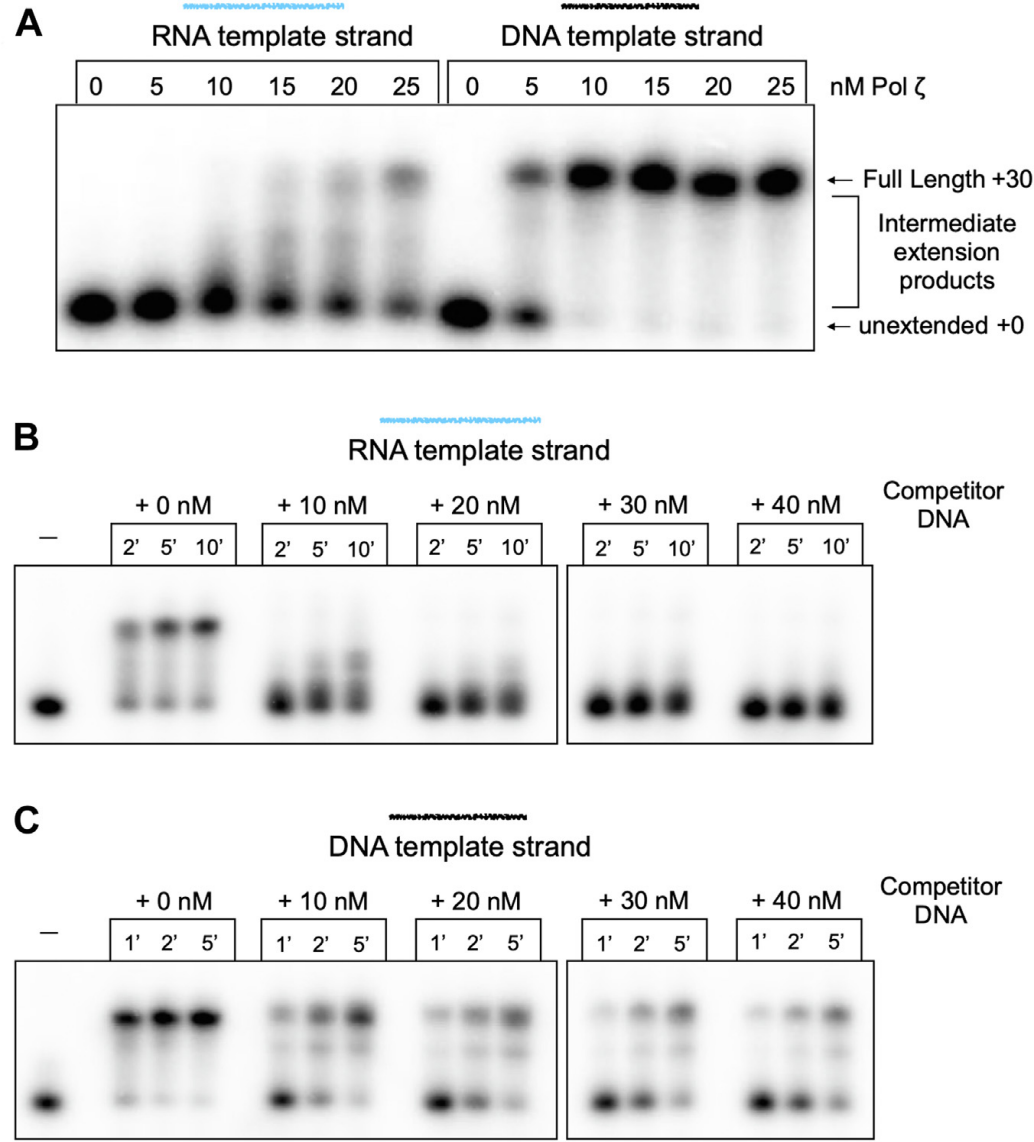
**Pol ζ is distributive during RT activity.***A*, titration of Pol ζ into extension assays with DNA primed RNA or DNA templates. RNA template reactions were stopped at 1 min and DNA template reactions at 10 s, and products were analyzed by denaturing PAGE. *B* and *C*, titration of competitor primed DNA into extension assays with DNA primed RNA (*B*) or DNA (*C*) templates. Aliquots of reactions were terminated at the indicated times followed by denaturing PAGE.

Next, we asked whether Pol ζ RT activity is processive or distributive in the context of extending 30 nucleotides to the end of our ^32^P-DNA primed RNA substrate. To determine this, we titrated Pol ζ into reactions containing a constant amount of DNA-primed RNA template. If Pol ζ were processive, the products would not show a dependence on the amount of Pol ζ added but would show more of the same product at different concentrations. If Pol ζ were distributive, the product length would depend on the concentration of the polymerase added. The results (Fig. 3*A*) show that product length depends on the concentration of Pol ζ, demonstrating that the reverse transcriptase activity of Pol ζ is distributive, and that processivity is less than 30 nucleotides in our assay conditions.

To further characterize the relative processivity of Pol ζ on DNA and RNA template strands, we performed primer extension assays with increasing concentrations of competitor primed DNA (Fig. 3, *B* and *C*). Similar to the Pol ζ titration experiment, increasing available substrate is expected to differentially impact product size for distributive vs processive synthesis. Distributive synthesis involves repeated rounds of polymerase turnover, and competitor DNA will therefore decrease the size of products, increasingly so at higher concentration of competitor. In contrast, processive synthesis will simply take longer to extend the increasing amount of substrate in the reaction while product lengths from our labeled substrate will not change. In other words, the pattern of products observed will remain the same for processive synthesis but the pattern of products will change for distributive synthesis as more and more competitor DNA is present. Consistent with the results in Figure 3*A*, we observed smaller products for the RNA substrate with increasing competitor, indicating distributive synthesis (less than 30 nucleotides). While we do not precisely resolve the length of individual rounds of extension, that length is substantially fewer than the 30 nucleotides required to generate full length product. Extension on the DNA substrate shows no difference in product lengths, consistent with an observed processivity of at least 30 nucleotides. There does appear to be a stall site in this substrate, which may be a sequence-dependent stall site or termination site. Regardless, the size of products does not change for DNA products, in sharp contrast to results using the RNA primed substrate.

**Figure 4:**
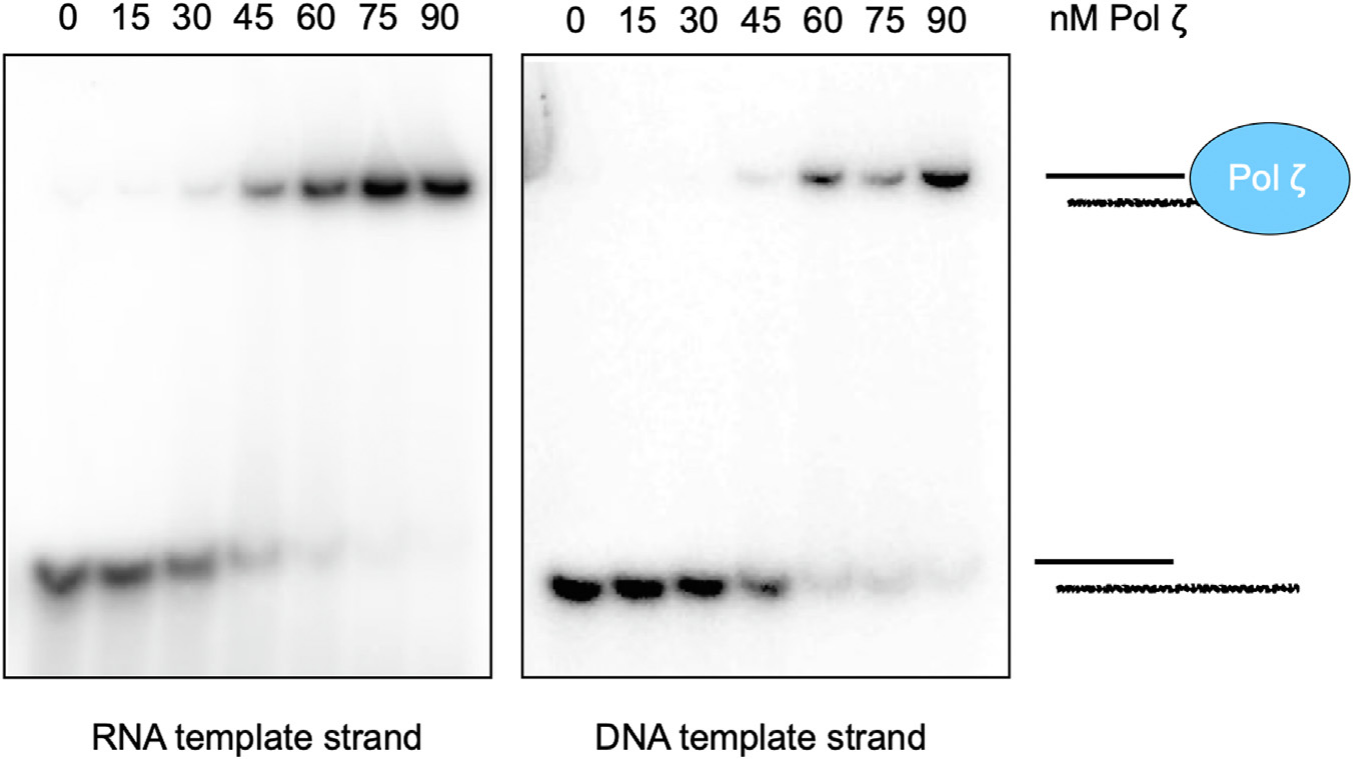
**Pol ζ has similar affinity for both RNA and DNA template strands.** Gel shift assay following incubation of Pol ζ with DNA primed DNA or RNA templates for 5 min at 30 °C.

One possibility for the observed differences in extension activity on DNA vs RNA template strands is that Pol ζ has a higher affinity for DNA than RNA. To address this, we performed an EMSA assay to compare binding affinity between Pol ζ and either DNA primed DNA or DNA primed RNA. Our results show that Pol ζ binds the RNA substrate as well, or better than the DNA substrate (Fig. 4). This suggests that differential binding affinity is not the primary factor in synthesis being slower on RNA, suggesting that the observed processivity (*i.e.*, Fig. 3, *C* and *D*) might explain this disparity. However another factor that could impact the observed difference in extension activity on RNA vs DNA is the catalytic rate of RT compared to DNA polymerase activity. Thus, either slower inherent polymerization, or greater distributive action (or both) may explain the lower activity of Pol ζ RT compared to DNA polymerase activity.

One may consider whether a distributive enzyme might be hindered by the single strand binding protein, RPA, which would undermine the view that Pol ζ might be a functional RT *in vivo*. To test this, we first examined binding of RPA to the RNA template primed with DNA under our primer extension assay conditions, by using an EMSA assay (Fig. S3). We observed that RPA binds the DNA primed RNA template when present at 100 nM, which matches the concentration used for RT assays performed in this report (Fig. S3).

Addition of RPA in extension assays of Pol ζ RT activity does not result in a significant inhibitory nor stimulatory effect (Fig. S4, *A* and *B*). We also tested the effect of adding RFC, PCNA, or RFC + PCNA to the Pol ζ RT assays (Figs. S4, *C* and *D* and S5*A*). We observed no notable inhibition or stimulation of Pol ζ RT activity by RFC and PCNA when added individually, or when added together. This could be due to RFC not loading PCNA on a DNA primed RNA template, consistent with a prior investigation of RFC’s loading abilities (26). We also observed that RFC + PCNA does not stimulate Pol ζ on a primed DNA substrate (Fig. S5*B*). Given these results, it may not be surprising that RFC + PCNA does not have an impact on RT activity.

## Discussion

There are several distinct pathways of DNA repair, as explained in the introductory material.. Among these pathways is a mechanism for DSB repair that uses the sequence of a transcript mRNA to regain any lost DNA information through the use of a reverse transcriptase. This can be accomplished by annealing of the RNA transcript across the DNA double-strand break followed by DNA fill-in using the mRNA transcript as a template (Fig. 1). Because this pathway was characterized in engineered yeast cells with multiple mutations, it remains to be seen how important this type of repair is in normally growing cells. However, what these genetic studies uncovered pointed directly to the idea that Pol ζ can act as a reverse transcriptase (13). While a yeast transposable element RT initially confused the issue, it was eventually shown that in its absence the chromosomal encoded genes that comprise Pol ζ were required for this method of DSB repair. Although the genetic studies suggested that Pol ζ might be the enzyme responsible for the reverse transcriptase activity, whether Pol ζ possessed RT activity had not been directly tested.

In this report, we purified each of the seven nuclear DNA Pols of *S. cerevisiae*. Upon examination of these different DNA Pols, we find that Pol ζ contains the most robust RT activity. Interestingly, Pol η is reported to contribute to RT synthesis relevant to DSB repair (18, 19). However, the relative RT activity of human Pols η and ζ is not known, and thus it remains to be determined whether the results of this report, using budding yeast Pols, generalize to human Pols. Importantly, Pol ζ has not been extensively studied in human cells in the context of RNA-templated repair and may possess similar activity to the budding yeast system explored here. Nonetheless, it is an exciting time for a deeper understanding of this newly discovered DSB repair pathway, and this report serves as a notable finding confirming the prediction of genetic studies that Pol ζ may possess RT activity (13). We hope to build on this work using recombination enzymes along with DNA Pol ζ and its associated machinery to reconstitute this transcription-based DSB repair process.

## Experimental procedures

### Proteins

Pol ε, Pol δ, Pol ⍺, RFC, RPA, and PCNA were purified as previously described (27). Each of Pol ζ, Pol η, Rev1, and Pol IV were cloned into the following integration vectors. Pol eta with an N-terminal 3× FLAG tag was cloned into pRS403 (His^+^ selection) under the control of the Gal1 promotor. N-terminal 3× FLAG-tagged Pol IV and Rev1 were each cloned into pRS404/GAL (Trp^+^ selection) under the control of the Gal1 promotor. The four subunit Pol ζ (Rev3/Rev7/Pol31/Pol32) was cloned into three integration vectors: Rev3 with a C-terminal 3× FLAG tag was cloned into pRS402/GAL (Ade^+^) under the Gal1 promotor. Rev7 was cloned into pRS405/GAL (Leu^+^) under the Gal1 promotor. Pol31 and Pol32 were cloned into the pRS403/GAL (His^+^) under the Gal1 and Gal10 promotors, respectively. The integration plasmids containing the genes of Pol η, Rev1, Pol IV, and the 4-subunit Pols ζ were integrated into yeast strain OY01 (27). Unlike other Pols, Pol ζ is four subunits and requires the integration of two plasmids instead of one, as noted above.

24 L of each strain encoding either Pol ζ, Pol η, or Pol IV was grown to 0.6 OD_600_ and induced with galactose for 6 h at 30 °C as previously described for Pols δ and α (28). The Rev1 strain was grown similarly, to 0.6 OD_600_, except was induced at 26 °C after only 4 h of Gal induction. The DNA Pols were purified similarly using the following protocol. 24L-induced yeast cells were lysed in a SPEX cryogenic mill as described (27). Lysed cells were clarified by centrifugation and 1 to 2 ml anti-flag beads (Sigma, #A2220) were added to the supernatant (about 150 ml), followed by 1 h on a rocking platform at 4 °C. The beads were then collected by centrifugation at 1500 rpm for 9 min in a Sorvall BP8 rotor at 4 °C. The supernatant was discarded and the beads were resuspended in 500 ml Buffer H (20 mM Hepes-Cl, pH 7.5, 1 mM EDTA, 10% glycerol, and protease inhibitors (Cytiva #P8210), followed by centrifugation at 1500 rpm for 9 min in a Sorvall BP8 rotor at 4 °C. This washing procedure was followed two more times, and then the beads were resuspended in 50 ml Buffer H, centrifuged once again, then resuspended in 5 ml buffer H, and applied to a “C column” (Cytiva). The column was then connected to an ACTA FPLC and rinsed with buffer H + 1M NaCl until the OD_280_ no longer changed (about 3 column volumes). Elution was with buffer H + 300 mM KCl plus 0.2 mg/ml 3×-FLAG peptide, using three 1-column volume pulses (30 min). Proteins were visualized in 10% SDS PAGE, pooled, and concentrated to 2 to 2.5 μM using a 30 kDa cut-off filter to remove FLAG peptide. Proteins were aliquoted and stored at −80 °C.

### DNAs

DNA oligonucleotide substrates were ordered from (Integrated DNA Technologies). Sequences used in this report are:

DNA Template Strand: TGT GGT AGG AAG TGA GAA TTG GAG AGT GTG GGT GAG GGT TGG GAA GTG GC.

RNA Template Strand: rUrGrU rGrGrU rArGrG rArArG rUrGrA rGrArA rUrUrG rGrArG rArGrU rGrUrG rGrGrU rGrArG rGrGrU rUrGrG rGrArA rGrUrG rGrC.

Primer: GTC TCG AGC CCA TCC TTC CAC TTC CCA ACC CTC ACC.

Biotin Primer: GTC TCG AGC CCA TCC /TEGBiotin/ TTC CAC TTC CCA ACC CTC ACC.

### Substrates

Oligo pair substrates were annealed in 100 mM Tris-Cl pH 7.5, 1 mM EDTA, and 100 mM KCl at a final concentration of 120 nM labeled primer with 180 nM unlabeled template strand to ensure saturation of the labeled primer. Two primary substrates were annealed, containing the primer and either the DNA or RNA template strand. Substrates were used at a final concentration of 1 nM in all reactions.

### Primer extension assay

Reactions were performed in reaction buffer (20 mM Tris-Acetate pH 7.5, 4% glycerol, 0.1 mM EDTA, 40 μg/ml BSA, 5 mM DTT, and 10 mM MgSO_4_). To initiate reactions, polymerase mixes containing the four dNTPs (100 μM final concentration each) and polymerase (20 nM final concentration, unless otherwise indicated) were added to substrate mixes containing either RNA or DNA template strand substrates, each primed with a 5′-^32^P end-labeled DNA oligo. Reactions were incubated at 30 °C for the indicated length of time. Positive control lanes are cM-MuLV RT (New England Biolabs) and negative control lanes are substrate mixes prior to polymerase addition. For reactions containing RFC-PCNA, a modified substrate was used in which the primer oligo has a TEGBiotin modification and the substrate was pre-bound with RPA to prevent loaded PCNA from sliding off the short substrate. For reactions containing RPA, substrate mixes with and without RPA (100 nM final concentration) were incubated for 5 min at 30 °C to allow RPA to bind the ssDNA before reactions were initiated upon the addition of polymerase. Reactions were terminated by adding an equal volume of 2× Stop buffer (1% SDS, 50 mM EDTA, 8% glycerol, 0.01% bromophenol blue, 83% formamide, 100 nM unlabeled primer oligo), boiled for 5 min, and analyzed in a 12% Urea PAGE gel. Gels were exposed to a phosphor imaging screen for 16 to 18 h then scanned with an Amersham Typhoon.

### Competitor DNA extension assay

Reactions were assembled as described above with the addition of 0, 10, 20, 30, or 40 nM unlabeled DNA-primed DNA substrate to the substrate mix before adding polymerase and dNTPs to initiate reactions. For the RNA template, 20 nM Pol ζ was used, and reaction aliquots were terminated at 2, 5, and 10 min. For the DNA template, 5 nM Pol ζ was used, and reaction aliquots were terminated at 1, 2, and 5 min.

### Gel shift assay

Reactions were set up in the same buffer conditions as in the primer extension assay described above. 100 nM RPA was incubated with 1 nM DNA primed RNA template for 5 min at 30 °C. One 10th volume of 10× loading dye (80% glycerol, 0.1% bromphenol blue) was added to each reaction, and samples were analyzed in a 10% native PAGE gel. For Pol ζ gel shift, 0 to 90 nM polymerase was incubated with 1 nM DNA primer DNA or DNA primer RNA for 5 min at 30 °C.

## Data availability

All data described in this report are contained within the article. Furthermore, all strains and plasmids of this report will be made available upon request.

## Supporting information

This article contains supporting information.

## Conflict of interest

The authors declare that they have no conflicts of interest with the contents of this article.
