# Supplementary material for: DNA polymerase ζ has robust reverse transcriptase activity relative to other cellular DNA polymerases
Source: J Biol Chem. 2024 Oct 23;300(12):107918. doi: 10.1016/j.jbc.2024.107918 (PMC11599448; doi:10.1016/j.jbc.2024.107918)
Supplement: Supplemental Figures S1–S5 [file mmc1.pdf]

DNA polymerase ζ has robust reverse transcriptase activity

Ryan Mayle[1], William K. Holloman[2]*, Michael E. O'Donnell[1]*

Supporting Information
Material Included:

Figure S1
Figure S2
Figure S3
Figure S4
Figure S5

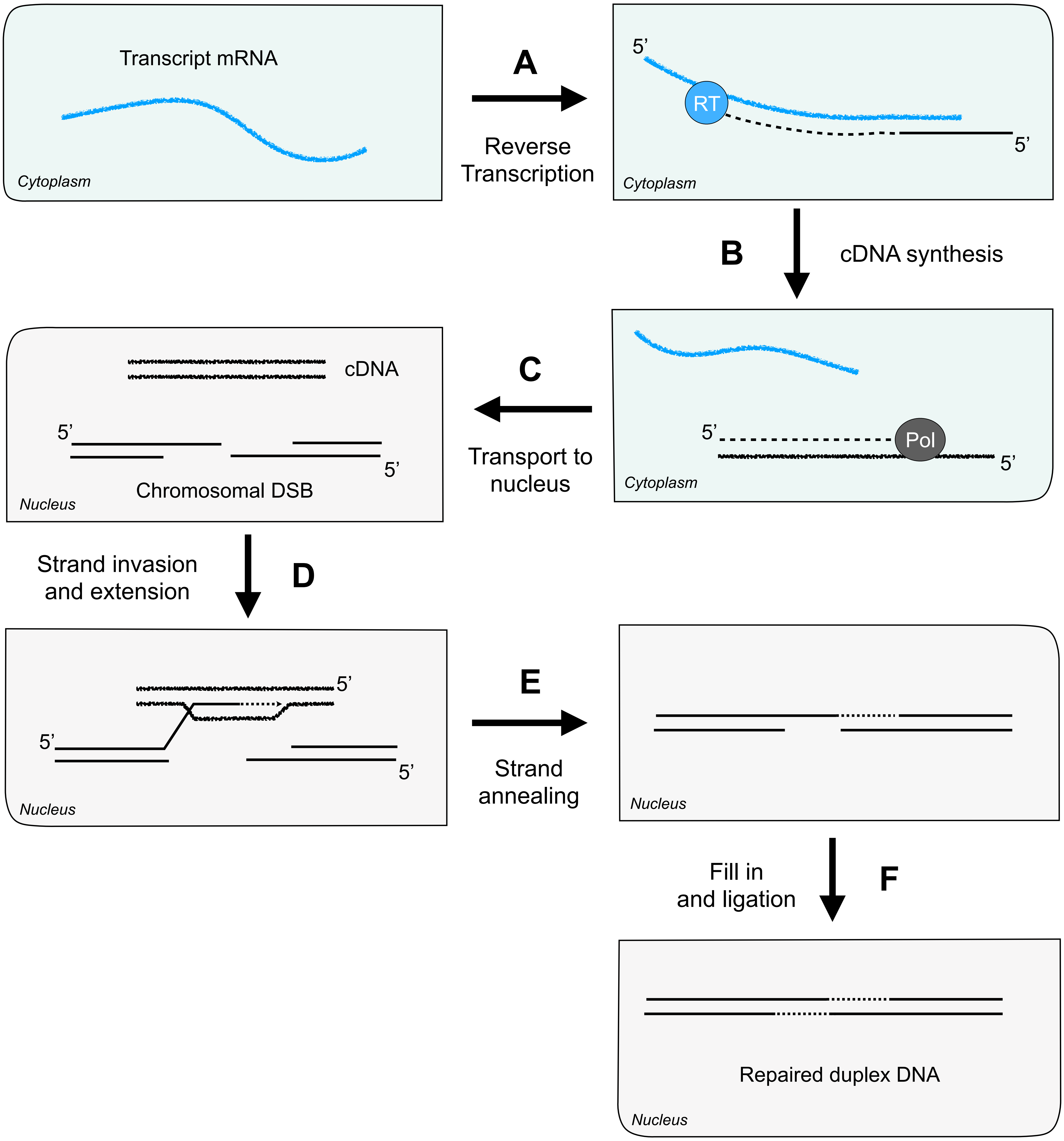

Figure S1: Model of RNA templated DSB repair in trans: **A)** First a transcript mRNA is primed via homology or microhomology, believed to involve a Ty retrotransposon. Reverse transcription via the Ty RT creates an initial DNA strand from the RNA template. **B)** Next cDNA is synthesized using the DNA strand created by the RT. **C)** This cDNA is transported back to the nucleus, where it can template repair of a DSB. **D)** Following resection of the DSB, strand exchange occurs and repair synthesis is templated by the cDNA. **E)** In yeast, the most common HR pathway is via synthesis dependent strand annealing, where annealing of the newly synthesized DNA to the other break end bridges the DSB. **F)** Finally, the remaining gap is filled in and ligation completes repair to re-form an intact repaired DNA duplex.

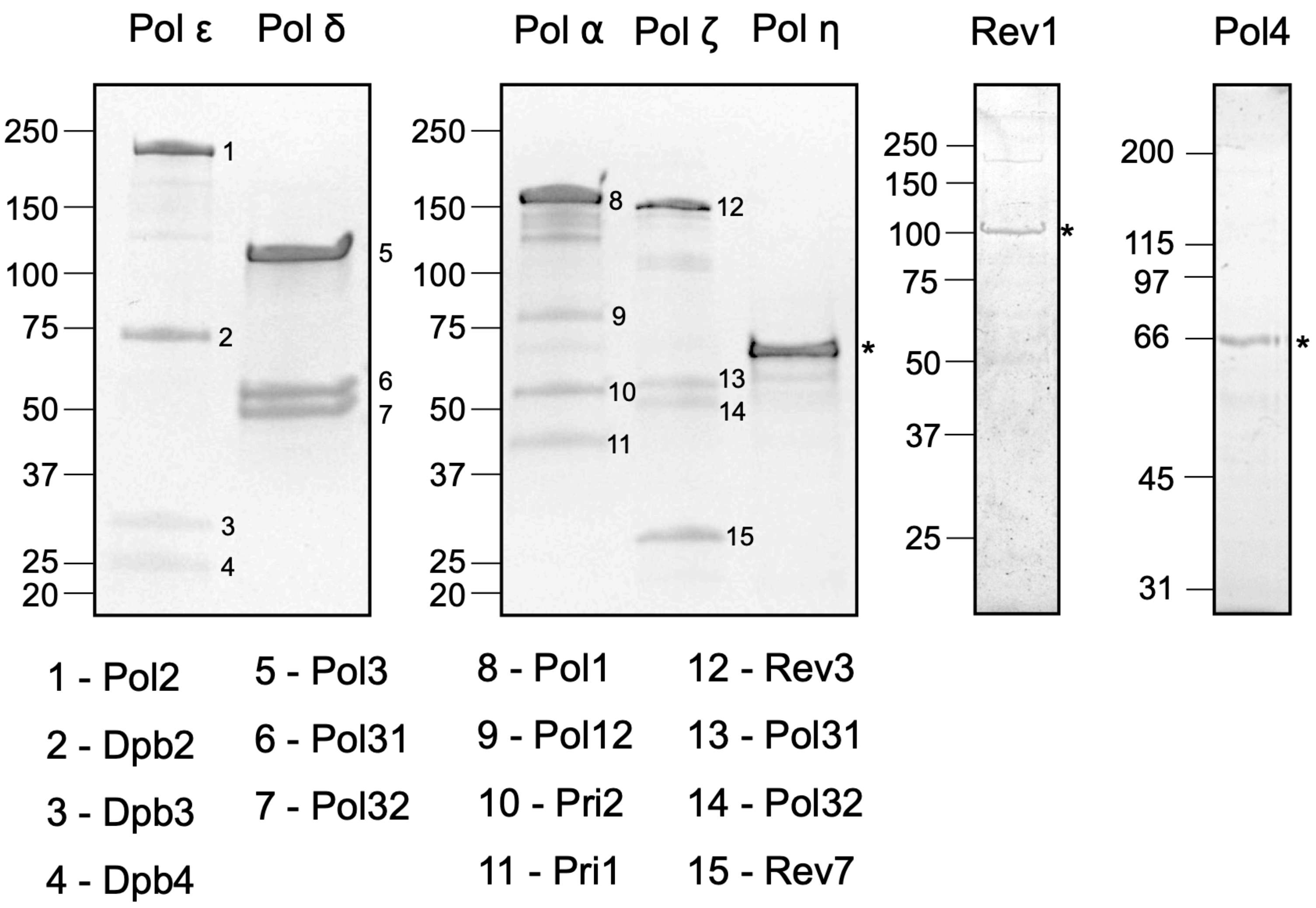

Figure S2: SDS-PAGE gels showing each of the purified yeast polymerases: Bands for individual proteins are indicated by either a number, for multi-subunit enzymes, or a * for single subunit polymerases.

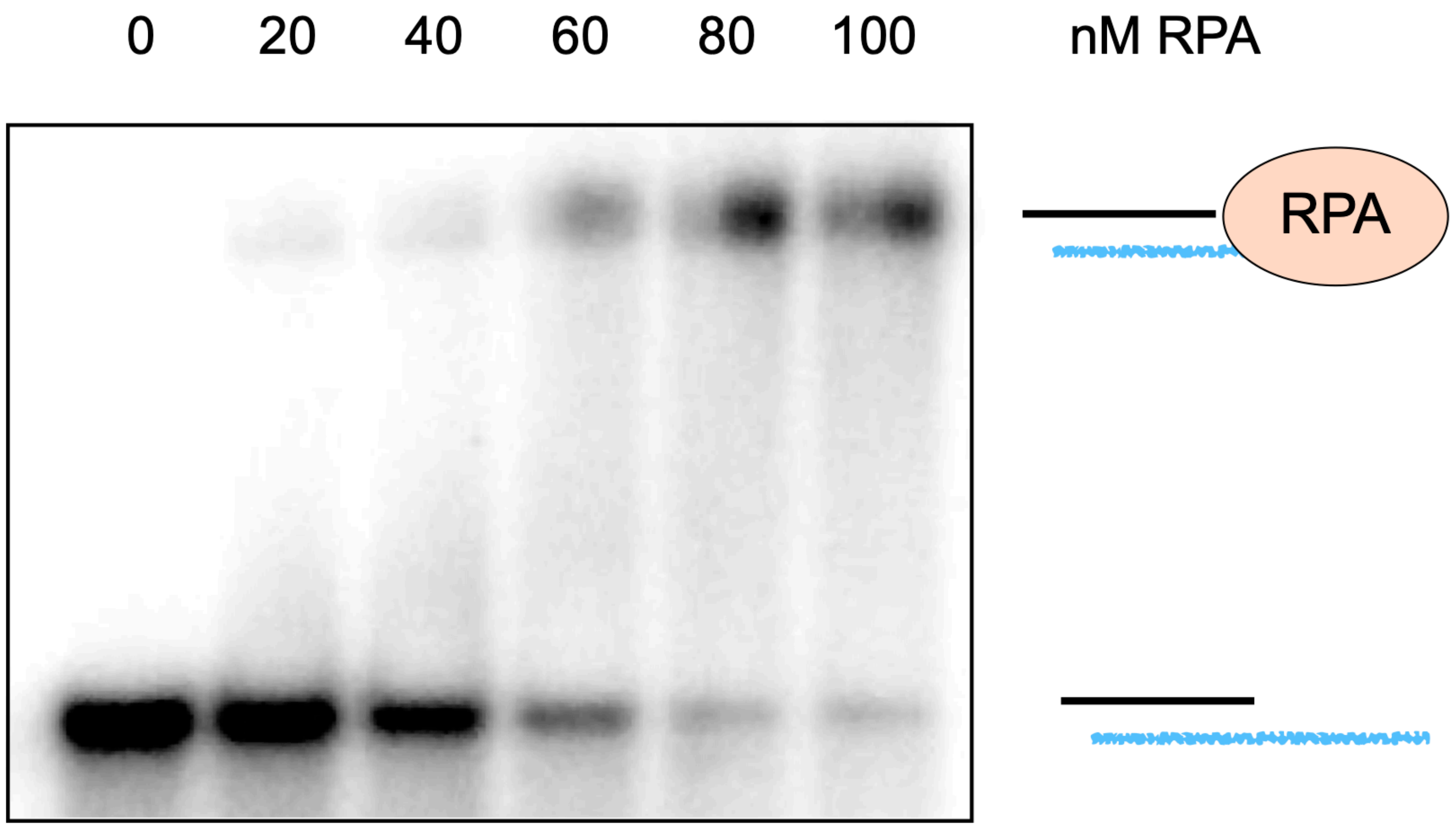

Figure S3. RPA binds the DNA primed RNA substrate: A gel shift assay after incubation of RPA with the DNA primed RNA substrate for 5 minutes at 30°C.

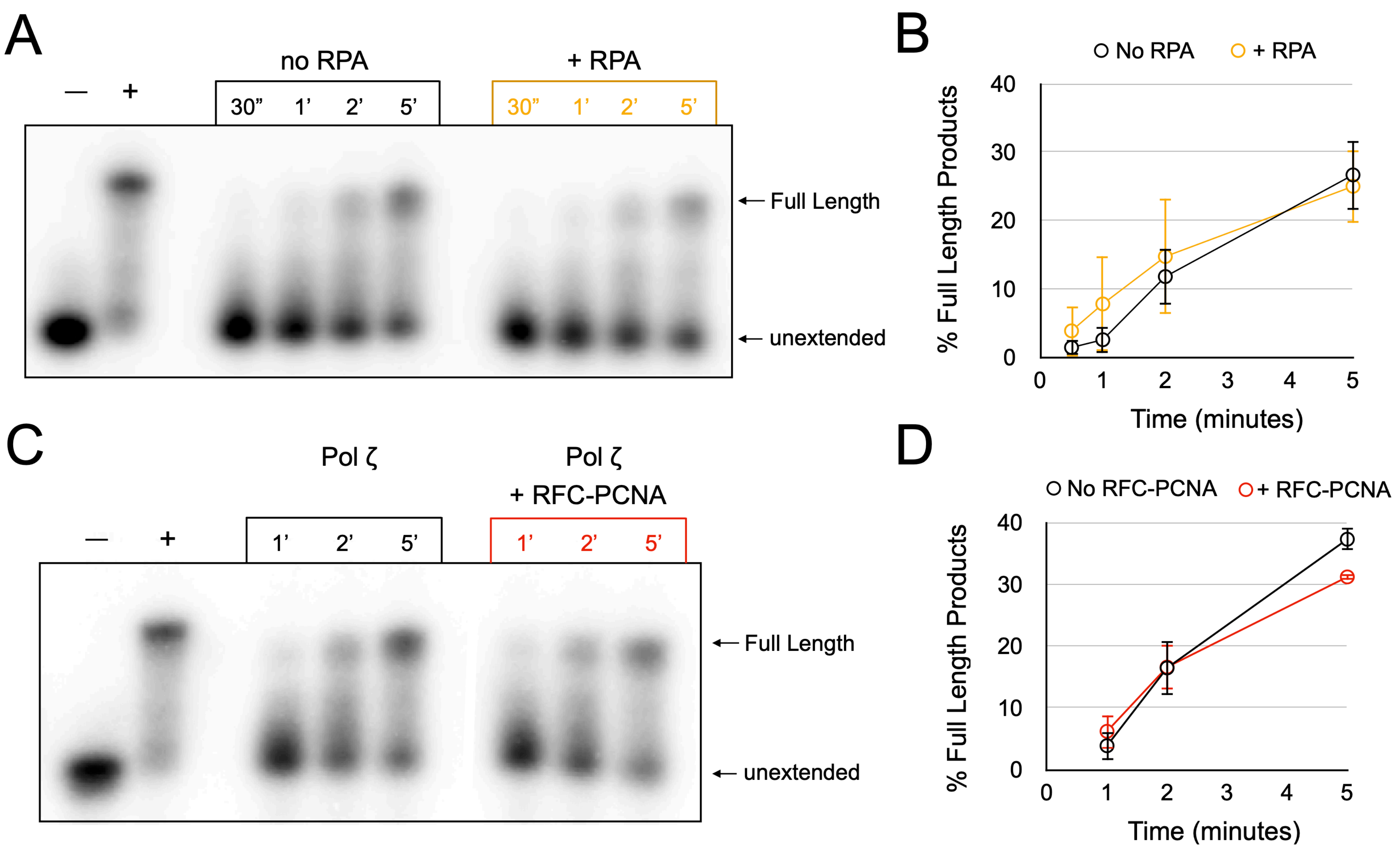

Figure S4. RPA and RFC/PCNA have no notable impact on Pol ζ RT activity: **(A)** shows primer extension RT assays comparing the presence and absence of RPA, and **(B)** is a quantitation of triplicate assays. Error bars represent +/- standard error of the mean. **(C)** shows primer extension RT assays comparing the presence and absence of RFC/PCNA. **(D)** is a quantitation of triplicate assays. Error bars represent +/- standard error of the mean.

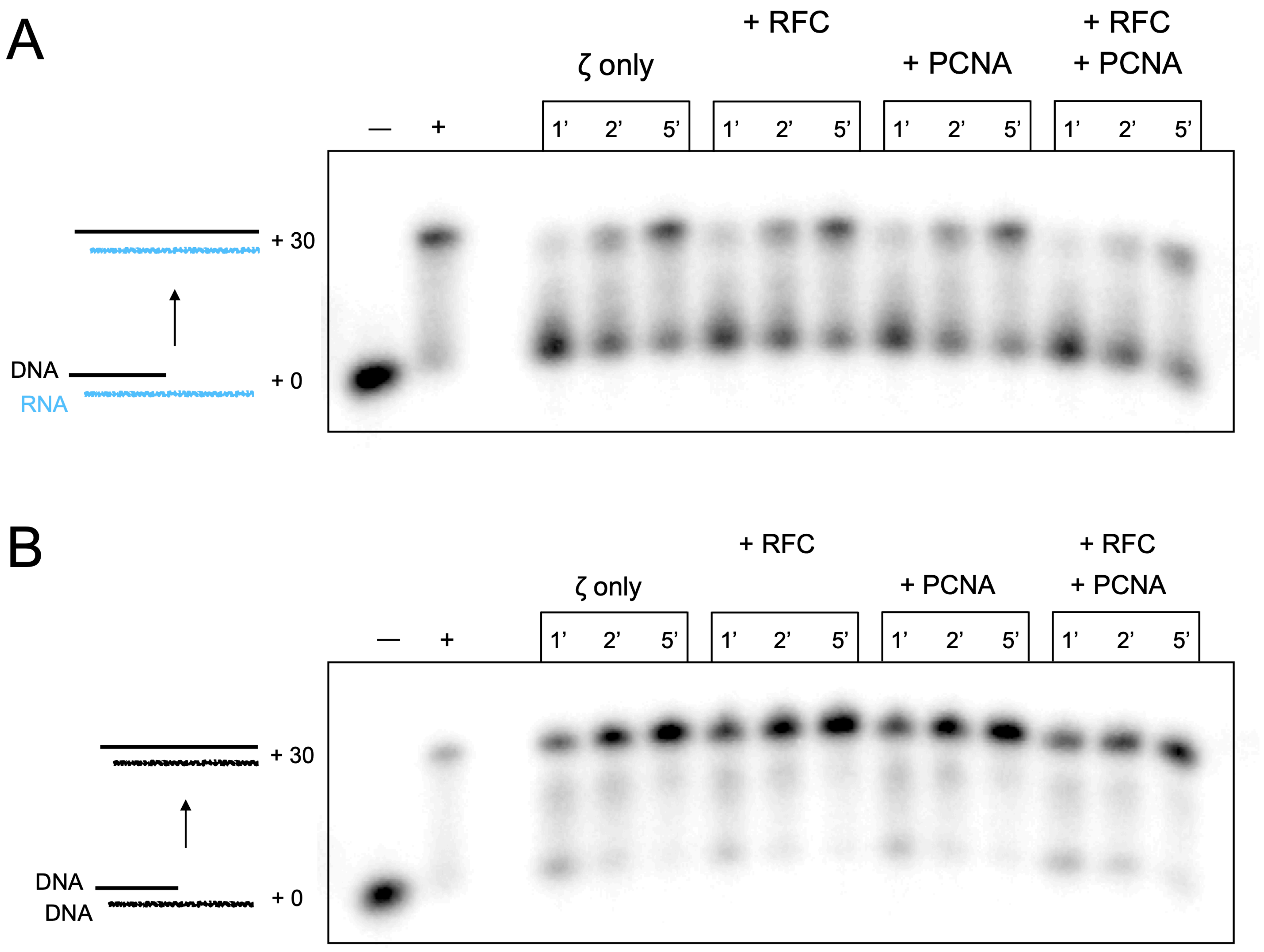

Figure S5: RFC +/- PCNA does not stimulate Pol ζ on either DNA or RNA templates: Extension assays with the noted additions of RFC and/ or PCNA using either RNA (A) or DNA (B) template strands.
